# Liquid Crystal Elastomer-Based Microelectrode Array for In Vitro Neuronal Recordings

**DOI:** 10.3390/mi9080416

**Published:** 2018-08-20

**Authors:** Rashed T. Rihani, Hyun Kim, Bryan J. Black, Rahul Atmaramani, Mohand O. Saed, Joseph J. Pancrazio, Taylor H. Ware

**Affiliations:** Department of Bioengineering, University of Texas at Dallas, Richardson, TX 75080, USA; Rashed.Rihani@utdallas.edu (R.T.R.); kimhyun@utdallas.edu (H.K.); bjb140530@utdallas.edu (B.J.B.); rxa162330@utdallas.edu (R.A.); Mohand.Saed@utdallas.edu (M.O.S.); Joseph.Pancrazio@utdallas.edu (J.J.P.)

**Keywords:** microelectrode array, liquid crystal elastomer, neuronal recordings

## Abstract

Polymer-based biomedical electronics provide a tunable platform to interact with nervous tissue both in vitro and in vivo. Ultimately, the ability to control functional properties of neural interfaces may provide important advantages to study the nervous system or to restore function in patients with neurodegenerative disorders. Liquid crystal elastomers (LCEs) are a class of smart materials that reversibly change shape when exposed to a variety of stimuli. Our interest in LCEs is based on leveraging this shape change to deploy electrode sites beyond the tissue regions exhibiting inflammation associated with chronic implantation. As a first step, we demonstrate that LCEs are cellular compatible materials that can be used as substrates for fabricating microelectrode arrays (MEAs) capable of recording single unit activity in vitro. Extracts from LCEs are non-cytotoxic (>70% normalized percent viability), as determined in accordance to ISO protocol 10993-5 using fibroblasts and primary murine cortical neurons. LCEs are also not functionally neurotoxic as determined by exposing cortical neurons cultured on conventional microelectrode arrays to LCE extract for 48 h. Microelectrode arrays fabricated on LCEs are stable, as determined by electrochemical impedance spectroscopy. Examination of the impedance and phase at 1 kHz, a frequency associated with single unit recording, showed results well within range of electrophysiological recordings over 30 days of monitoring in phosphate-buffered saline (PBS). Moreover, the LCE arrays are shown to support viable cortical neuronal cultures over 27 days in vitro and to enable recording of prominent extracellular biopotentials comparable to those achieved with conventional commercially-available microelectrode arrays.

## 1. Introduction

Neural interfaces allow for communication with nervous tissue both in vitro and in vivo. In vitro neural interfaces, such as planar microelectrode arrays (MEAs), allow for characterization of cultured neural networks, which is effective in a variety of in vitro model applications such as neuropharmacological applications and cell compartmentalization [1,2,3]. The use of polymers for in vitro neural interfaces has proven advantageous and gained traction over the past years [3,4,5,6]. This includes the fabrication of mechanically flexible planar MEAs to reduce the tissue-interface mechanical mismatch [7] or using polymer actuators to allow for advanced interface control in the case of cell compartmentalization [8]. In vivo neural interfaces, such as implantable microelectrode arrays, offer a means of functional restoration in patients who suffer paralysis, strokes, limb loss, or neurodegenerative disease [9]. However, the reliability of such neural interfaces is compromised, in part, by the body’s own foreign body response (FBR), leading to localized astrogliosis and fibrotic encapsulation of the device [10]. These factors may lead to accelerated mechanical/electrical device failure and/or loss of neurons at the site of implantation [11,12]. While conventional implantable microelectrode arrays are comprised of inherently stiff materials, flexible polymer substrates have gained interest for their potential in mitigating the mechanical mismatch at the tissue–device interface and reduce FBR-induced encapsulation [13,14]. 

Biostable and biocompatible polymer packaging may provide important advantages over traditional ceramic devices including mechanical flexibility and compatibility with tissue. These polymer-based hybrid devices consist of a metallic electrode array and a polymeric package [15]. It is critical that the chosen polymer also form a sufficient barrier to insulate the electronics from the moist physiological environment [16]. Various types of polymers—such as polyimide [17], Parylene [18], shape memory polymers [19], and liquid crystal polymer (LCP) [15,20]—have been tested as compliant, insulating materials in neural interfaces. Particularly, LCPs have gained significant attention as a promising substrate material for long-term implantable neural interfaces due to its low moisture absorption, which is equivalent to that of polytetrafluoroethylene (PTFE) and glass (<0.04%) [21,22]. This absorption is significantly lower than those of other polymers such as polyimide (~2.8%), Parylene-C (0.06–0.6%) and silicone elastomers (~1%) [20]. Certain classes of LCPs may offer additional functionality beyond serving as a robust barrier, including by enabling deployable neural interfaces. 

Most polymer-based bioelectronic devices are planar in nature, as photolithography is used to fabricate the devices. Three-dimensional and reconfigurable bioelectronic systems may enable devices that dynamically adapt to external physiological environments [23]. To go beyond the capability of current static devices, we aim to integrate shape-changing materials and microelectronic devices into a single dynamic reconfigurable system. LCEs [24,25,26], a subclass of LCPs, contain light crosslinking density and tunable transition temperatures which allows for a reversible shape change in response to a stimulus such as heat [27,28], light [29,30], or solvent [31,32]. This approach potentially enables the controlled deployment of small recording or stimulation electrode sites to regions beyond that of the FBR-induced tissue encapsulation zone surrounding an implanted shank (50–100 μm) [33,34,35,36,37]. Recently several publications have suggested that some compositions of LCEs may be cytocompatible [38,39,40]. However, there have been no prior studies evaluating cytotoxicity, neurotoxicity, or manufacturability of LCE as a functional electrode array package. Notably, the performance of LCEs as barriers to the physiological environment has not been evaluated. Here, we apply International Organization for Standards (ISO) protocol 10993-5 to evaluate the cytotoxicity of LCEs using both NCTC clone 929 fibroblasts and primary murine cortical neurons. Additionally, we evaluate the functional neurotoxicity of LCE materials in vitro using primary neuronal networks cultured on commercially available planar microelectrode arrays. Finally, we report the fabrication and characterization of functional planar MEAs using LCEs as an insulating package material. 

## 2. Materials and Methods 

### 2.1. Fabrication of LCE MEAs

**Figure 1:**
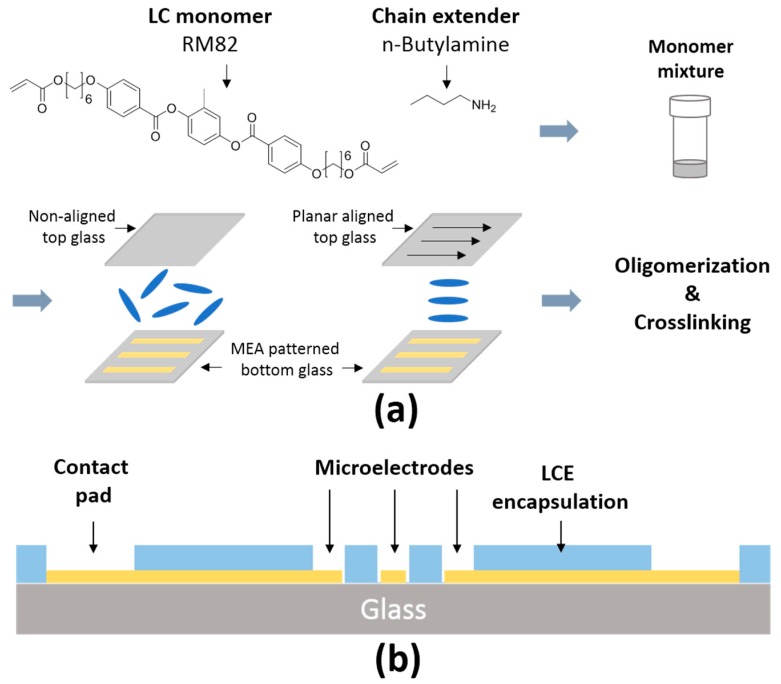
Chemistry of LCE and MEA fabrication: (**a**) Molecular structure of monomers used to synthesize the LCE and device fabrication procedure; (**b**) Cross-sectional schematic of LCE MEA devices.

Figure 1a,b depict the fabrication of the MEA and a schematic of the layers that comprise the MEA. To fabricate MEAs, microscope glass slides (75 mm × 51 mm × 1.2 mm, Electron Microscopy Sciences, Hatfield, PA, USA) were serially cleaned with acetone, isopropanol, and deionized water and subsequently dried with nitrogen (Figure 1). Then, 5 nm of chromium and 400 nm of gold were serially deposited via e-beam evaporation (Temescal BJD-1800, Ferrotec Corporation, Livermore, CA, USA). The deposition rate in this process was set to 2–3 Å/s. After coating of the Cr/Au to the glass slides, a positive photoresist (Shipley S1805, Dow Chemical, Midland, MI, USA) was spun at 2000 rpm with an acceleration of 3000 rpm/s for 60 s and soft baked at 85 °C for 12 min. The photoresist was exposed to 75 mJ/cm^2^ of UV light using a Karl Suss MA6 Mask Aligner (SÜSS MicroTec, Garching, Germany). Photoresist development was performed in Microposit MF-319 (Dow Chemical, Midland, MI, USA) for 60 s. Etching of the Au was performed using gold etchant (Transene Company, Midland, MI, USA) for approximately 60 s. Etching of the Cr was performed using chrome etchant (KMG Electronic Chemicals, Pueblo, CO, USA) for approximately 10 s. The remaining photoresist was removed by a flood exposure of 150 mJ/cm^2^ UV light and subsequent development in MF-319. 

To encapsulate MEAs with LCEs, a previously described chemistry was used [27,31]. The liquid crystal monomer, 1,4-bis-[4-(6-acryloyloxyhexyloxy)-benzoyloxy]-2-methylbenzene (RM82), was purchased from Wilshire Chemicals (Princeton, NJ, USA). The chain extender molecule, n-butylamine, was purchased from Sigma Aldrich (St. Louis, MO, USA). The photoinitiator, Irgacure I-369, was donated by BASF (Ludwigshafen, Germany). The photoalignable dye, brilliant yellow, was purchased from Sigma Aldrich. To prepare the top side glass slide, a solution of Brilliant yellow (1 wt. %) in dimethylformamide (DMF, Fisher Scientific, Pittsburgh, PA, USA) was prepared and filtered through a 0.45 μm filter (Whatman, Maidstone, UK). Clean glass slides were treated with oxygen plasma for 1 min at 100 mTorr pressure and 50 mW power (Sirius T2, Trion Technology, Tempe, AZ, USA). The brilliant yellow solution was then spin-coated onto the cleaned slides at 750 rpm with an acceleration of 1500 rpm/s for 10 s and 1500 rpm with an acceleration of 1500 rpm/s for 30 s. A previously described photoalignment procedure was adopted [41]. Dye-coated glass slides were exposed to broadband, linearly polarized light at an intensity of 10 mW/cm^2^ (Vivitek D912HD, Vivitek, Fremont, CA, USA). A pair of glass substrates, one with the patterned electrodes and one coated with the dye (uniaxial alignment or non-aligned) were spaced 25 μm apart using a spacer (Precision Brand, Downers Grove, IL, USA) along the edges. This mold was filled with a monomer mixture of an equimolar amount RM82 and n-butylamine mixed with 1.5 wt. % of photoinitiator I-369. The monomer mixtures were filled by capillary force and kept for 15 h at 65 °C for oligomerization. Then, oligomerized samples were crosslinked with 250 mW/cm^2^ intensity of 365 nm UV light (OmniCure^®^ LX400+, Lumen Dynamics, Mississauga, ON, Canada) for 5 min. After crosslinking, the top-side glass slide was removed yielding an electrode array covered with a layer of LCE. The optical images of LCE MEA devices were observed by a polarized optical microscope (POM) (Olympus BX51, Olympus Corporation, Tokyo, Japan). 

To finalize fabrication of LCE MEA devices, the fully insulated MEAs were coated by 800 nm silicon nitride at 150 °C using Plasma Enhanced Chemical Vapor Deposition Unaxis 790 PECVD (Mykrolis Corporation, Billerica, MA, USA). Then hexamethyldisilazane (HMDS, Sigma Aldrich, St. Louis, MO, USA) was vapor-deposited onto the silicon nitride to serve as an adhesion layer for photoresist. A positive photoresist (Shipley S1813, Dow Chemical, Midland, MI, USA) was spun at 500 rpm for 10 s with an acceleration of 100 rpm/s and 2000 rpm for 60 s with an acceleration of 3000 rpm/s and soft baked at 85 °C for 12 min. The photoresist was exposed to 150 mJ/cm^2^ of UV light using a Karl Suss MA6 Mask Aligner. The silicon nitride was patterned using dry etching by 120 mTorr pressure and 100 mW power with SF_6_ (Sirius T2, Trion Technology, Tempe, AZ, USA). Next, the encapsulated LCE layer was patterned via dry etching by 220 mTorr pressure and 200 mW power with oxygen plasma. The remaining hard mask was removed with a 60 s rinse of hydrofluoric acid (1:10) (HF) treatment. Lastly, a polycarbonate ring (0.6 cm height, 2.0 cm inner diameter, and 2.2 cm outer diameter) was attached to the surface of the LCE MEA devices with a silicone adhesive (MED1-4213, Nu-Sil, USA) as described in a previous study [5].

### 2.2. Electrochemical Characterization of LCE MEAs

All devices were sterilized by exposure to ethylene oxide prior to testing. Electrochemical impedance spectroscopy (EIS) measurements were carried out on eight randomly selected electrodes from LCE microelectrode arrays for 30 consecutive days in PBS at 37 °C. For impedance measurements, the experimental setup consisted of a three-electrode configuration (working, ground, reference), wherein the external MEA pads were used to deliver a sinusoidal 20 mV signal, and impedance was measured by a CH 604E potentiostat (CH Instruments, Austin, TX, USA) between 10 Hz and 100 kHz. Between measurements, MEAs were housed in a cell culture incubator at 37 °C and 95% humidity between measurements. Fresh PBS was replaced prior to each measurement to account for any evaporation and subsequent osmolarity changes. The sample size of four MEAs decreased to three MEAs starting on day 24 due to inadvertent manual damage to one of the MEAs.

### 2.3. In Vitro Cytotoxicity Testing

All handling, housing, and surgical procedures of the mice were approved by the University of Texas Institutional Animal Care and Use Committee. Cytotoxicity assays were carried out as previously described [42] and in accordance with the ISO protocol “10993-5: Biological evaluation of medical devices” using both NTC 929 fibroblasts (ATCC, Manassas, VA, USA) and embryonic day 15 (E15) mouse-derived cortical neurons. Briefly, 50 and 100% concentration LCE extract was evaluated against Tygon-F-4040-lubricant tubing extract (positive control) and cell medium (negative control) [42]. In accordance with the ISO protocol, materials were said to ‘pass’ if normalized cell viability percentages exceeded 70% following 24-h incubation with material extracts.

Cortices were surgically dissected from E18 mouse embryos, dissociated, and cultured as previously described [4,5]. Prior to seeding, 24 well polystyrene plates (Greiner Bio-One, Kremsminster, Austria) were treated with 50 μg/mL poly-d-lysine (PDL) (Sigma-Aldrich, Saint Louis, MO, USA) and 20 μg/mL laminin to facilitate cell adhesion. Cells were seeded at a density of 100,000 cells/well and incubated at 37 °C, 10% CO_2_, and 95% humidity in proliferation medium (Dulbecco’s Modified Eagle Medium, GlutaMAX, B-27, ascorbic acid, and 10% horse serum). Serum was reduced to 0% over the span of 5 days to avoid over-proliferation and ganglionation of supporting cells. Fibroblasts were sub-cultured prior to seeding in a 24 well polystyrene plate. Cells were incubated at 37 °C, 10% CO_2_, and 95% humidity in complete medium (Dulbecco’s Modified Eagle Medium and 10% horse serum). Material extracts were made by soaking strips of LCE (3 cm^2^/mL) in normal cell medium (Dulbecco’s Modified Eagle Medium) at 37 °C, 10% CO_2_, and 95% humidity for 24 h. When cells formed a semi-confluent layer, cell medium was exchanged for 50% or 100% material extract concentrations. Cells were incubated in extract for 24 h prior to using a LIVE/DEAD cytotoxicity kit for mammalian cells according to manufacturer’s protocol (Thermo Fisher, L3324, Waltham, MA, USA). Briefly, cells were stained with 2 μM Calcein-AM and 4 μM Ethidium homodimer for live and dead cells, respectively. Images were collected using a 10× objective on an inverted microscope (Nikon Ti eclipse, Nikon, Tokyo, Japan). Cell counts were carried out using a boutique ImageJ (NIH, Bethesda, MD, USA) macro, which applied a 2.0 Gaussian blur before locating local intensity maxima. Cells that were stained with both dyes were marked as “dual-stained” and were regarded as part of the dead cell count based on a MATLAB code.

### 2.4. In Vitro Functional Neurotoxicity Testing

Functional neurotoxicity assays were carried out as previously described [42]. Briefly, primary cortical neurons were seeded on 48 well Axion well plates (Axion Biosystems, Atlanta, GA, USA) after treating wells with 50 μg/mL PDL and 20 μg/mL laminin. On DIV 23, baseline spontaneous extracellular activity was recorded using Axion’s Maestro recording system (Axion, Atlanta, GA, USA). Immediately following baseline recordings, cell medium was exchanged for LCE extract at concentrations of 50% or 100%. Spontaneous extracellular activity was recorded again on DIV 25. Spike and burst rates were determined using a custom MATLAB script. An active electrode was defined as an electrode exhibiting more than five spikes per minute. Inactive electrode sites recorded on DIV 23 were excluded from any further analysis. A burst was defined as at least five consecutive spikes with interspike intervals less than 100 ms [43].

### 2.5. In Vitro Neuronal Recordings and Pharmacology

Three polydomain and planar-aligned LCE MEAs were sterilized by ethylene oxide for 12 h, and then de-gassed at 37 °C for 48 h. LCE MEAs were treated as described in Section 2.3. Every other day, wide band extracellular potentials generated by cultured neurons were recorded for 5 min from 59 channels simultaneously at a 40 kHz sampling rate using an Omniplex data acquisition system (Plexon Inc., Dallas, TX, USA). Wideband data were band pass filtered (250–7000 Hz) and spikes were detected by voltage excursions exceeding a threshold set to 5.5σ based on RMS noise on a per electrode basis. Spikes were manually sorted using Plexon’s offline sorter (Plexon Inc., Dallas, TX, USA). Additional data and statistical analyses were carried out using OriginPro software (Origin Labs, Farmington, ME, USA). Average spike rates were calculated using Neuroexplorer (NEX technologies, Reston, VA, USA). SNR was calculated as

(1) $$\mathrm{SNR}=(\frac{Signal}{RMS_{Noise}}),$$

where Signal and $RMS_{Noise}$ are the mean peak-to-peak amplitude of the sorted unit and the RMS noise, respectively [43]. Active electrode yield percentage was calculated excluding electrodes with impedances over 5 MΩ that would not be capable of recording single units [5]. As a result, 8.2% of total electrodes were excluded.

### 2.6. Statistical and Data Analysis

All experiments were carried out in parallel using both polydomain and planar-aligned LCEs. This was done to determine if the alignment procedure itself affected any changes on the stability of the substrate. However, these groups are, in fact, chemically identical, and we observed no apparent differences between the groups in any of the results reported here. Therefore, these results derived from both polydomain and planar aligned have been pooled in the following sections.

All statistical analyses were carried out using OriginPro software (Origin Labs, Farmington, ME, USA). In the case of functional neurotoxicity tests, treatment groups were compared using a two-sample *t*-test. In the case of TTX treatments on LCE MEAs and electrochemical stability, a paired two-tailed *t*-test was applied. *p* < 0.05 was considered statistically significant in all cases.

## 3. Results

To investigate the use of LCEs as substrate materials for neural interfaces, we examine the cytotoxicity, functional neurotoxicity, and manufacturability of microelectrodes on LCEs. Here, we use ISO protocol 10993-5 to evaluate the cytotoxicity of LCEs using both fibroblasts and primary murine cortical neurons. Additionally, we evaluate the functional neurotoxicity of LCE materials in vitro using primary murine cortical neurons cultured on commercially available planar MEAs. Finally, we report the fabrication and characterization of functional planar MEAs using LCEs as an insulating layer. 

### 3.1. In Vitro Cytotoxicity Testing

**Figure 2:**
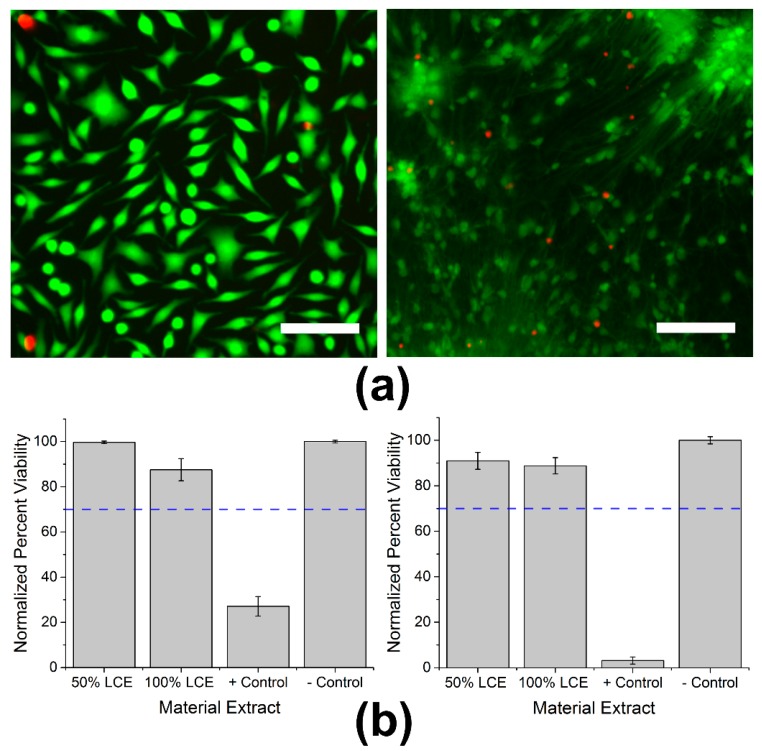
Cytotoxicity assays using both NCTC fibroblasts and primary cortical neurons: (**a**) Fluorescent images of NCTC fibroblasts (**left**) and primary murine-derived cortical neurons (**right**) stained with CaAM (green) for live cells and EthD-1 (red) for apoptotic cells. Scale bars represent 100 μm (**b**) Mean normalized fibroblast (**left**) and cortical neuron (**right**) viability percentages of LCE extract at 50% and 100% concentrations, positive control, and negative control. Blue dotted line at 70% represents the ISO threshold set for non-cytotoxic materials.

To determine whether LCEs induce cytotoxicity, live/dead assays were carried out in accordance to ISO protocol 10993-5 using both NTC fibroblasts and primary mouse-derived cortical neurons. Cells were stained with both live and dead markers after exposure to extract as seen in Figure 2a. The threshold for in vitro cytotoxicity is a normalized viability of greater than 70% after extract exposure. After 24 h of exposure to 50 or 100% LCE extracts, NTC fibroblasts exhibited normalized viability percentages of 99.7 ± 0.6 and 87.6 ± 4.9% (mean ± SEM, n = 5). On exposure to positive control material extract (Tygon tubing), NTC fibroblasts exhibited a significantly lower normalized viability percentage of 27.1% ± 4.3 (mean ± SEM, n = 5, *p* < 0.0001). The normalized viability of primary cortical neurons with exposure to 50 or 100% concentrations of LCE extracts was 91.0 ± 3.7 and 88.8 ± 3.6% (mean ± SEM, n = 5), respectively. In contrast, exposure to Tygon tubing (positive control) extract resulted in a statistically significant reduction in normalized viability percentage to 3.2% ± 1.6 (mean ± SEM, n = 5, *p* < 0.0001) (Figure 2b). While exposure to 100% LCE extracts caused a significant reduction in normalized viability percentage to both fibroblasts (*p* = 0.04) and cortical neurons (*p* = 0.02) when compared to the negative control, these values were well above the 70% threshold set by ISO standards, suggesting that the LCE material is not cytotoxic to either NTC fibroblasts or primary cortical neurons. 

### 3.2. In Vitro Functional Neurotoxicity Testing

**Figure 3:**
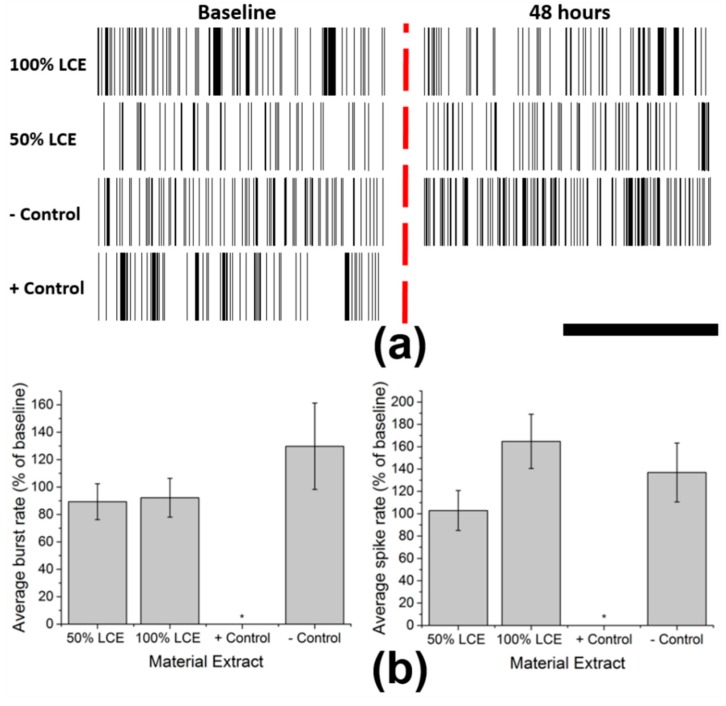
Functional neurotoxicity assays using primary cortical neurons: (**a**) Representative raster plots of spontaneous activity from single electrodes before (**left**) and after (**right**) each treatment. Scale bar represents 3 s. (**b**) Average Burst rate (**left**) and spike rate (**right**) of cortical neurons after 48 h exposure to LCE extract at 50% and 100% concentrations, positive control, and negative control.

While it is paramount to maintain cell viability in the presence of LCE devices, it is equally important that LCE neural interfaces ultimately not affect neuronal network activity. To determine whether neuronal network activity is modulated in the presence of LCE material extracts, we measured mean firing rates and bursting rates in the presence and absence of 50% or 100% material extract treatments using cortical neurons cultured on commercially-available MEAs. After 21 days in culture, neuronal network activity reached a plateau with a mean spike rate of 2.72 ± 0.55 Hz (mean ± SEM) and a burst rate of 0.75 ± 0.11 Hz (mean ± SEM) (n = 19 wells). On DIV 23, a 10 minute baseline recording was taken, then medium was exchanged for LCE extract at 50% and 100% concentrations. Parallel experiments were performed with positive (Tygon extract) and negative control (cell medium) treatments. A second recording was taken to compare to baseline after 48 h of exposure. Exposure of primary cortical neurons to LCE extracts did not significantly change neuronal network parameters (Figure 3a). Normalized mean spike and burst rates after exposure to positive control extracts were significantly reduced to 0% ± 0 (mean ± SEM) (n = 4) (*p* = 0.003 for spike rate, *p* = 0.008 for burst rate). LCE extracts failed to significantly alter bursts (*p* = 0.27 at 50%, 0.31 at 100%) or spike rates (*p* = 0.32 at 50%, 0.46 at 100%) when compared to the negative control, suggesting functional biocompatibility (Figure 3b). 

### 3.3. Electrochemical Impedance Stability of MEAs

**Figure 4:**
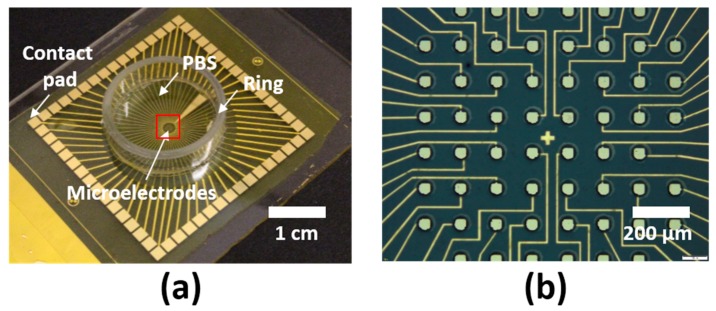
LCE encapsulated MEA devices: (**a**) Optical image of LCE encapsulated MEA device with polycarbonate ring attachment. Red square indicates microelectrode sites at the center of the device. (**b**) Reflection optical microscope image of the microelectrode site. 30 × 30 μm microelectrodes are exposed by 50 μm windows for recording of extracellular activity. Electrical traces are fully encapsulated with LCE.

After evaluation of cytotoxicity and functional neurotoxicity, LCE MEAs (Figure 4a,b) were fabricated as described above. Initial impedances ranged from 248.7 kΩ to 627.1 kΩ at 1 kHz. To examine the electrochemical stability in LCE MEAs, MEAs were aged at 37 °C in PBS for 30 days. The initial recorded impedance magnitudes of electrode sites ranged from 248.7 kΩ to 627.1 kΩ at 1 kHz on day 0 and averaged 381.7 kΩ ± 22.3 (mean ± SEM, n = 32 electrodes, 8 each from 4 MEAs). The final recorded impedance magnitude at 1 kHz on day 30 was 120.3 kΩ ± 42.7 (mean ± SEM) (n = 24 electrodes, 8 from each MEA). While this change was statistically significant (*p* < 0.05), the decrease was relatively minor and was largely attributed to an initial decrease over days 0–4 followed by a prolonged period of apparent stability. The recorded phase on day 0 at 1 kHz was −53.9° ± 1.6 (mean ± SEM) (n = 32 electrodes, 8 from each MEA). The final recorded phase at 1 kHz on day 30 was −68.5° ± 0.6 (mean ± SEM) (n = 24 electrodes, 8 from each MEA). There was no significant trend found in the phase at 1 kHz over the span of 30 days (*p* = 0.47, ANOVA). Even though there is a significant change in the impedance magnitude at 1 kHz through the 30 days, the final recorded impedance at 1 kHz is still well within the range of impedances capable of recording physiological signals, suggesting that LCE MEAs are sufficiently electrochemically stable for neural interfaces [4,5,44]. 

### 3.4. In Vitro Neuronal Recordings and Pharmacology

**Figure 5:**
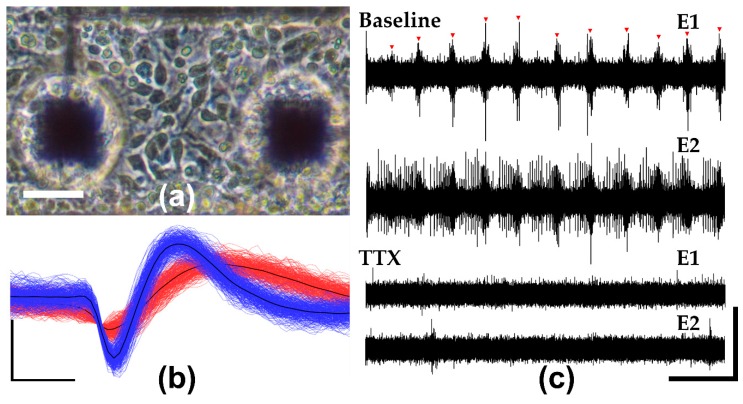
Neuronal Recordings and pharmacology on LCE MEAs: (**a**) Phase contrast image of cultured cortical neurons on LCE MEA DIV2. Scale bar represents 30 μm. (**b**) Representative extracellular waveforms recorded from LCE MEAs on DIV 21. Vertical and horizontal scale bars represent 40 μV and 280 μs. (**c**) Representative bandpass-filtered extracellular recordings from 2 representative electrodes on a single MEA (DIV 27) before and after application of TTX. Red arrowheads indicate network bursts. Vertical and horizontal scale bars represent 55 μV and 2 s.

To assess the neural recording capabilities of LCE MEAs, six MEAs were seeded with primary embryonic mouse-derived cortical neurons and recorded over the course of 27 days in vitro. Figure 5a shows that LCE MEAs supported cell adhesion as can be seen by the phase-dark cell bodies and minimal cell clumping on DIV 2 (Figure 5a). Five of the six arrays showed excellent adhesion and network activity and were selected for further analysis.

Using LCE MEAs, extracellular action potentials could be readily detected and sorted into collections of characteristic waveforms (single units, Figure 5b). By DIV 23, single units were detected on 56 ± 8% (mean ± SEM, n = 5) of electrode sites with a mean SNR of 8.9 ± 0.8 (mean ± SEM, n = 5). To confirm that the recorded spikes were biologically sourced, cortical cultures were exposed to 100 nM of TTX, a sodium channel blocker, on DIV 27. In the presence of TTX, mean spiking rates were significantly reduced from 2.8 ± 0.5 to 0.5 ± 0.2 Hz (mean ± SEM, n = 4) based on a paired *t*-test (*p* = 0.03), suggesting that recorded activity from MEAs was physiological (Figure 5c). Overall, mature cortical networks on DIV 23 exhibited a mean spiking rate of 2.7 ± 0.9 (mean ± SEM, n = 5), and mean bursting rate of 4.5 ± 1.5 (mean ± SEM, n = 5), suggesting development of synaptically-driven network activity. These values are all highly consistent with those reported in previous studies using commercial MEAs [4,42].

## 4. Discussion

The goal of this study is to examine the cellular compatibility of LCE as a material to serve for novel neural interfaces and to demonstrate the manufacturability of the material for fabricating devices. LCEs are a class of smart materials that undergo reversible changes in shape on exposure to a variety of stimuli [33,34,35,36,37]. No previous study has evaluated the suitability of LCE as a substrate and/or insulating material for microelectrode arrays for neural interface applications. Here, we have demonstrated that material extract treatment assays, carried out in accordance with ISO protocol 10993-5, do not indicate significant cytotoxicity in cultures of either NTC fibroblasts or primary cortical neurons; material extract treatments do not significantly modulate primary cortical network activity; that LCE-encapsulated in vitro MEAs exhibit stable impedance measurements over 30 days at 37 °C; and that MEAs could be fabricated, sterilized, and used to record primary neuronal activity for 27 days in vitro. In total, these results suggest that LCE may be a viable substrate for designing and fabricating neural microelectrodes for in vitro and, potentially, in vivo applications. Such devices may provide new capabilities, including deployable microelectrodes.
